# Dyspnea in Patient with Arteria Lusoria: A Case Report

**DOI:** 10.22038/ijorl.2020.46502.2525

**Published:** 2020-09

**Authors:** Nicola Massaro, Barbara Verro, Giuseppe Greco, Carmelo Saraniti

**Affiliations:** 1 *Department of Biomedicine, Neurosciences and Advanced Diagnostic, University of Palermo, Palermo, Italy.*

**Keywords:** Arteria lusoria, Dysphagia, Dyspnea, Vascular anomaly

## Abstract

**Introduction:**

Arteria lusoria is an aberrant right subclavian artery. In symptomatic cases, patients report dysphagia and only in few cases dyspnea, due to external compression of the trachea and esophagus. Symptoms occur in advanced age and diagnosis is made with chest HRCT, when other causes of dysphagia have been excluded.

**Case Report:**

An 83-year-old woman presented with dyspnea and mechanical dysphagia for solids. Therefore, she did a chest high-resolution computed tomography (HRCT) that showed areas of consolidation of the lung parenchyma, pleural effusion and presence of arteria lusoria, with a retroesophageal course. After 18 days, dysphagia and dyspnea worsened. The new chest HRCT revealed bilateral atelectasis of the lower lung lobes and severe compression of esophagus and trachea along the course of the arteria lusoria.

**Conclusion:**

Considering its dangerousness, this vascular anomaly should be considered in advanced aged patients with dysphagia and dyspnea, once other causes have been excluded.

## Introduction

The presence of an aberrant right subclavian artery, called arteria lusoria, may cause dyspnea and dysphagia because this vessel can compress externally the trachea and esophagus, respectively. Arteria lusoria is considered aberrant because it does not arise from the brachiocephalic trunk (as it is physiological) but from the aortic arch, laterally to the left subclavian arch. After its origin, the vessel crosses the median line and reaches the right side. As for its location, arteria lusoria can be posterior to the esophagus (80% of cases), between the trachea and esophagus (15% of cases) and anterior to the trachea (in 5% of cases) (1). The presence of this aberrant artery is rare (2) and it is most frequently asymptomatic. In symptomatic cases, patients report above all dysphagia and only in few cases dyspnea (3). Moreover, in elderly patients these symptoms can be more severe due to greater rigidity of the vascular wall of the aberrant artery or due to the formation of aneurysms along its course (4,5). Patients may also have other symptoms such as: cough, retrosternal pain and weight loss (5). Chest HRCT is essential for a certain diagnosis, once other causes of dysphagia have been excluded. The therapeutic approaches include diet modification or vascular surgery in order to restore physiological vascularization (5).

Although lusoria dysphagia and dyspnea are rare conditions, they should be considered in the differential diagnosis once other organic causes have been excluded, above all if patients are of advanced age, because of its potential dangerousness.

## Case Report

An 83-year-old woman suffering from acute myeloid leukemia for two years and chronic obstructive pulmonary disease (COPD) was referred to our attention. She presented with dyspnea due to the exacerbation of COPD, bilateral pleural effusion and mechanical dysphagia for solids. At the time of admission to the hospital, her blood tests showed high inflammatory indices, thrombocytopenia and normochromic anemia. Moreover, her body temperature was 39 degrees Celsius and she needed oxygen therapy at a flow rate of 5 L/min. On admission, due to her symptoms, she did a chest HRCT (high-resolution computed tomography) that showed areas of consolidation of the lung parenchyma, pleural effusion and presence of arteria lusoria (or aberrant right subclavian artery), with a retroesophageal course, responsible for dysphagia (^Fig’s 1^,2). She also did endoscopy of the upper aero-digestive tract that was normal. She immediately started antibiotic therapy.

**Fig 1:**
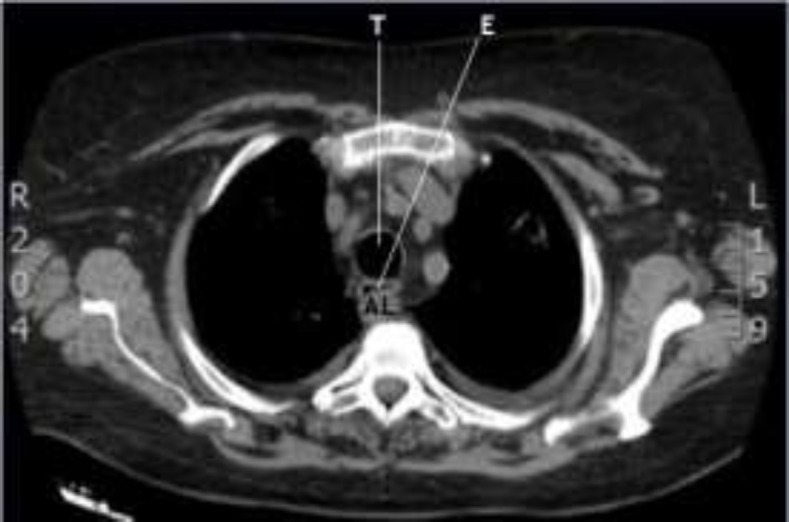
HRTC, axial section (T=trachea, E= esophagus, AL = arteria lusoria)

**Fig 2:**
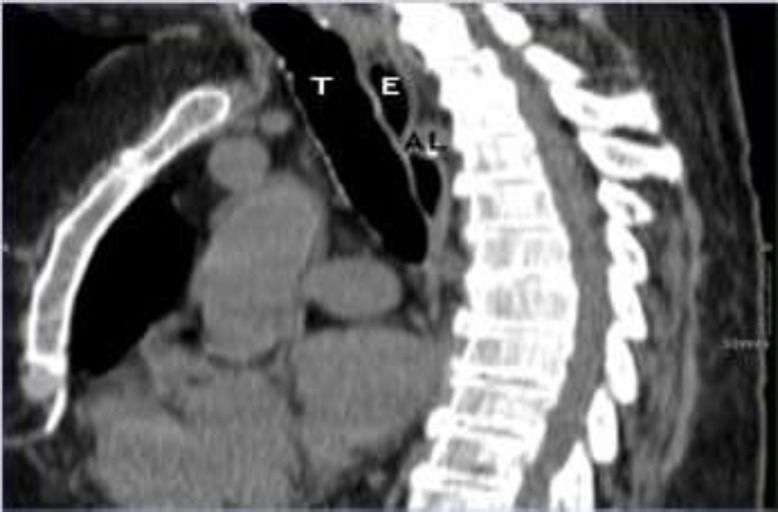
HRCT, sagittal section (T= trachea, E= esophagus, AL = arteria lusoria)

Six days after admission, the patient did another chest HRCT that revealed a reduction of the pleural effusion with an unchanged pulmonary status and substantial stability of the trachea. (^Fig’s 3^,4).

**Fig 3:**
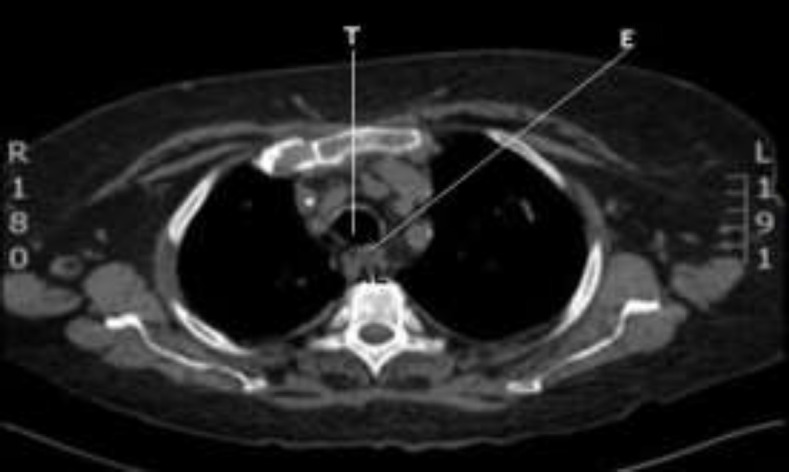
HRTC, axial section (T= trachea, E= esophagus, AL = arteria lusoria)

**Fig 4:**
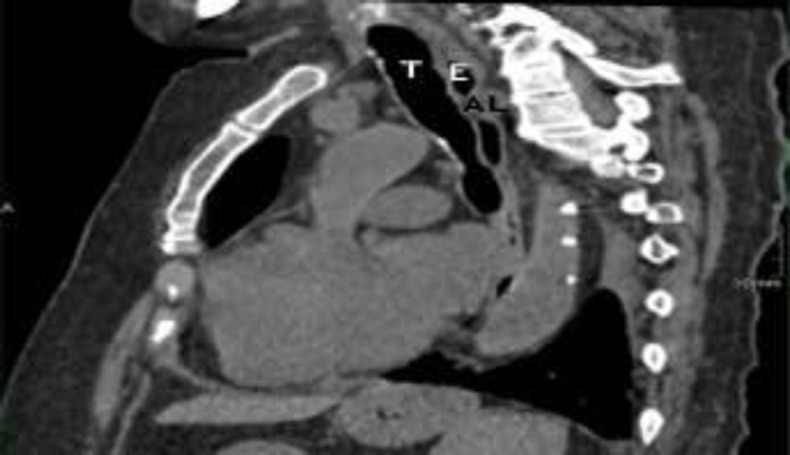
HRCT, sagittal section (T = trachea, E = esophagus, AL = arteria lusoria)

After another twelve days, the patient showed a sudden worsening of dysphagia for solids and liquids and dyspnea: so, she did an urgent brain and chest HRCT. Intra- or extra-axial blood spills and recent onset of ischemic lesions were excluded. Chest HRCT revealed bilateral atelectasis of the lower lung lobes and severe compression of the esophagus and trachea. The caliber of trachea was reduced along the course of arteria lusoria. (^Fig’s 5^,6).

**Fig 5:**
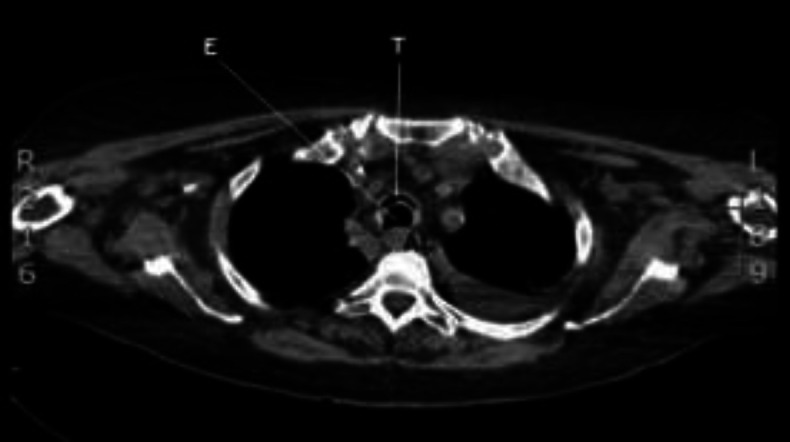
HRCT, axial section (T = trachea, E = esophagus, AL = arteria lusoria)

**Fig 6:**
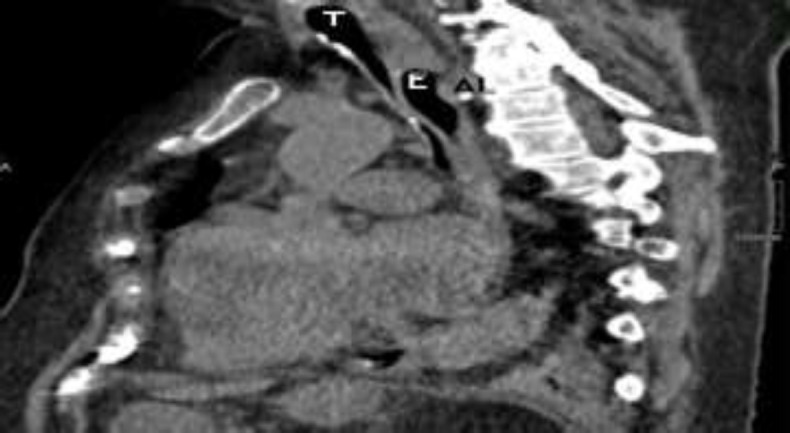
HRCT, sagittal section (T = trachea, E = esophagus, AL = arteria lusoria)

Due to the patient’s important comorbidities and the pulmonary clinical status, thoracic and vascular surgeons advised against surgical strategy. Therefore, the patient was intubated and transferred to the ICU for close monitoring. She died 24 hours later due to severe respiratory failure.

## Discussion

Lusoria dyspnea and dysphagia are caused by extraluminal compression of the trachea and esophagus, respectively, by an aberrant right subclavian artery, called lusoria. This vessel does not originate from the brachiocephalic trunk (as it normally does) but from the aortic arch, laterally to the left subclavian arch and then reaches the contralateral side crossing the median line. In 80% of cases, arteria lusoria is posterior to the esophagus, in 15% of cases it is between the trachea and esophagus and in 5% it is anterior to the trachea (1). It has an incidence between 0.4% and 0.7% (2). 

Compression symptoms occur in advanced age in 20% to 40% of patients (2,6). Indeed, this condition is most frequently asymptomatic. In the literature, dysphagia is reported in more than 90% of symptomatic cases (6), while dyspnea is reported in few cases (3). 

In very elderly patients, symptoms may appear more severe because of the increased rigidity of the vascular wall of the aberrant artery, resulting from atherosclerosis or fibromuscular dysplasia, or after the formation of aneurysms along its course (4,5). Other related symptoms may be cough, retrosternal pain and weight loss (5). 

A certain diagnosis is made with chest HRCT, when other causes of dysphagia have been excluded. Moreover, video fluoroscopic swallowing exam can help in the diagnosis because it may show extrinsic compression of the thoracic esophagus. 

Therapy consists in appropriate dietary modifications or vascular surgical procedures that interrupt the aberrant vascularization and restore the physiological one (5). Surgical therapy is strongly influenced by the patient’s age and comorbidities: indeed, in our clinical case, the patient could not receive surgery due to her old age and comorbidities and dyspnea lusoria was decisive for the prognosis quoad vitam.

## Conclusion

Although lusoria dysphagia and dyspnea are rare, they should be taken into account in differential diagnosis once other organic or neurological causes have been excluded, especially when these symptomatic conditions occur in advanced age.
